# On the Connection Subsisting between the Gout and Hypochondriasis in Men, and the Gout and Hysteria in Women

**Published:** 1799-04

**Authors:** 

**Affiliations:** Copenhagen


### *On the Connection subsisting between the Gout and Hypochondriasis in Men, and the Gout and Hysteria in Women: By Professor* Tode, *of Copenhagen.*

(Extract from the Journal of Inventions, Theories, and Controversies in Medical and Natural Philosophy, Gotha, 1798.

According to the opinion of the celebrated Prof. Tode, of Copenhagen, " hypochondriasis is merely an imperfect podagra residing in the stomach and bowels, whereas its natural and proper situation is in the great toe." This idea, though not a little eccentric, and even whimsical at first view, the ingenious author supports with many plausible arguments. In a small work which he has lately published, intitled : " *Necessary instructions for hypochondriacs who wish to become acquainted with their real situation, and guard themselves against danger;* 8vo. Copenhagen, 1797." (in German) the Professor reasons chiefly upon the following facts, 1. That a similar bodily constitution renders persons equally disposed to hypochondriasis, as it does to the gout; 2. that hypochondraical symptoms frequently alternate with attacks of the podagra, and that immediately on the perception of the latter, the former cease and disappear. Upon the strength of these two facts, Dr. Tode is anxious to establish the identity of what are commonly considered separate, the hypochondriasis and the podagra. With due deference to this learned writer, however, we cannot assent to this doctrine ; for the following reasons. 1. With respect to the alledged similarity of bodily constitutions, subject to the attacks of the hypocondriasis and podagra ; this analogy either proves too much, namely, the identity of all diseases to which persons of a robust constitution * are subject; or too little, as it is well known, that *real* hypochondriacs are far from possessing a sound healthy constitution. And although we were to admit the alteration between the attacks of the hypochondriasis and those of the podagra, this would not establish the identity of the two diseases. It is a fact generally admitted by medical practitioners, that one distemper may remove or suppress the other, in a variety of ways, without any identity subsisting between the two, and without any change or translation of a morbid matter from the place of the first to that of the second disorder. The gout, therefore, may probably silence the complaints and sufferings of the hypochondriac, by a sort of counter-revolution effected in the body. Thus, it has been observed, that after the expiration of cutaneous tumours, the gout or consumption followed; but can it on that account be justy inferred, that a cutaneous tumour

---

* According to Prof. Tode, persons afflicted with gouty and hypochondriacal diseases, generally possess a sound constitution.

tumour is virtually nothing but a concealed gout or consumption, limited to a certain part of the body? Thus, also, after eruptions or ulcers had been healed, the patient frequently became subject to epileptic fits; but can we, with any propriety, call such eruptions and ulcers, a cutaneous epilepsy? This, indeed, could not be consistently asserted; neither can the position of Mr. Tode be maintained, that the hypocondriasis is virtually the same thing with the gout in the stomach and bowels.

Much truth, however, is concealed in this unlimited doctrine of the Professor. The whole misconception in the proposed theory arises from the author's confounding *pure hypocondriasis* with a defective digestion, or *dyspepsia,* which is essentially connected with the arthritic disease, and which always, sooner or later, precedes the symptoms of the gout. This species of dyspepsia has been accurately described by Trampel, in his "*Observations and Experiments;* two volumes 8vo. 1788 and 1789. Lemgo:" (in German): and the partizans of the *Brunonian System* have very lately, though with much exultation and pretension to novelty, excited the attention of medical practitioners to that latent enemy of the gouty and hypochondriacal. This dyspepsia should be substituted for the hypochondriasis, in all cases where Mr. Tode speaks of its cure and connection with the gout. With respect to the *pure* hypocondriasis, we may safely appeal to the judgment of experienced medical men, that it is by no means necessarily connected with the podagra, and that real hypocondriacs are least subject to the gout; on the contrary, this latter phenomenon is seldom, if ever, observed by practitioners. It must however be admitted, that even the dyspepsia, as combined with the podagra, is very frequently a troublesome and torturing malady, and that it may be easily confounded with the hypocondriasis; particularly in times and countries, where it is not unfashionable to complain of hypochondriasis, and where only a small portion of *ennui,* discontent, ill humour, or a corrupted stomach, may suffice to procure a person the reputation of a complete hypocondriac.

Although Dr. Tode's pathology is inaccurate, with regard to *pure* hypochondriacs, yet the diet and regimen he prescribes will in general be found salutary; the medicines, however, which he recommends for the cure of the disease, are not only inadequate, but they are likewise too inert, and do not correspond with the leading indications to be attended to in the hypochondriasis. Whoever has had the treatment of patients of this description intrusted to his care, in whose stomachs there is a continual disposition to acidity, will neither hope nor expect to vanquish such a distemper, by administering a little magnesia and quassia, as Mr. Tode advises in his treatise. It is not however our intention to enlarge, in this place, upon the treatment of the the true hypochondriaſis, but only to remark, that the author has not accurately diſtinguiſhed this diſorder, which he appears to have confounded with dyſpepſia, the inſeparable companion of the gouty.

To afford the reader an opportunity of judging how far the *method of cure* preſcribed by the Profeſſor coincides with his theoretical notions, we ſhall here inſert, from the practical part of his eſſay, a few particulars relative to the medical and dietetical treatment of hypochondriacs.

" A patient, afflicted with hypochondriaſis, ought not to imagine that medicine will afford him much relief, or that nature requires chiefly artificial aid ; he may, however, expect more benefit from a proper attention to diet, than from dabbling in phyſic. It is an ill-founded conceit, that the numerous and diverſified ſymptoms of this diſeaſe require a tedious, artificial, and compound method of cure : on the contrary, as all theſe ſymptoms originate from the ſame ſource, a few appropriate medicines are ſufficient to anſwer the purpoſe of checking and ſuppreſſing them.

" I adviſe only one general method of curing this diſeaſe, which is uſually effectual in all its ſtages, and in all its ſymptoms ; as being well calculated to remove the prediſpoſing cauſe of the diſorder.—From whatever cauſe hypochondriaſis may ariſe, whether from nervous debility, or from a concealed podagra, it always depends on two different deviations from a ſtate of health, namely, firſt, *weakneſs* of the ſtomach or the inteſtines, as well as the other parts of the body ; and ſecondly, on an *acid* in the ſtomach.

" Weakneſs requires ſtrengthening remedies, and for this purpoſe the quaſſia is one of the moſt proper: acidity muſt be abſorbed, and to accompliſh this, the white magneſia is fully adequate.

" A third indication of cure ſometimes claims the attention of the practitioner, viz. to remove *ſpaſms*, which frequently occur in hypochondriac patients, although they are, in general, known only from their effects. Theſe require the uſe of one remedy only, namely, what is vulgarly called the nervous tincture *(liquor nervinus)*, being a ſolution of camphor in Hoffmann's anodyne liquor, which conſiſts of ambergris diſſolved in a ſpirit diſtilled over roſes, ſo that the patient take from half a grain to a grain of camphor for a doſe, frequently repeated. This powerful remedy ought, however, to be uſed only when the two preceding medicines have proved inefficacious, and after the firſt paſſages have been duly evacuated of acrimonious and acid humours, or at leaſt a proper regard paid to this important circumſtance."

With reſpect to *diet*, both as to the articles of food and drink, which are the moſt proper for hypochondriacs, Mr. Tode ſuggeſts many valuable hints.

Theſe occupy his 16th and 17th chapters, which extend to upwards of twenty pages, and of which, for want of room, we ſhall give the reader only a ſummary account.

The author juſtly deprecates the uſe of all acid and acidulated food and drink in this tedious diſorder. Of the vegetable articles, he recommends particularly, the ſweet red turnip, or beet-root, carrots, cauliflower, French beans, green peas while young and tender, mealy potatoes well boiled, lettuce, or other ſalad, without oil, vinegar, or eggs, ſweet French prunes, rice boiled in water, &c. On the contrary, he ſtrongly diſſuades hypochondriacs from the uſe of onions and garlick, as well as all other heating ſpices, cabbage, (even *ſour-kraut*, the favourite diſh of the Germans, which agrees only with thoſe who are not troubled with acidity,) all decoctions of oats and barley, as well as porridge, haſty puddings, diſhes made of meal, as paſtry, confectionary articles, and every kind of milk and cheeſe.— Among the different ſpecies of animal food, he conſiders beef, mutton, light game, and freſh fiſh, as wholeſome to hypochondriacs, but condemns veal and lamb, particularly when very young and tender, as being unwholeſome: beef or veal a-la-mode, is, according to him, extremely pernicious, on account of the quantity of ſpice employed in the cooking of theſe diſhes, which is but too frequently uſed for the purpoſe of covering or palliating a bad taſte, ariſing from the putrefaction already commenced in ſuch meat as is employed by public cooks; diſhes, which the late celebrated Dr. ZIMMERMAN emphatically ſaid, were of *infernal cookery*. Our author further inculcates the neceſſity of abſtaining from ſmoked and dried fiſh, eels, ſprats, ſalmons, crabs, lobſters, and herrings; the laſt of which, however, may be eaten ſalted, with moderation, if not too fat.

In the article of beverage, he prefers claret-wine to all others; of the ſweet wines, Madeira is the moſt conducive to the health of hypochondriac and nervous patients; but Rheniſh wine and Champaigne are hurtful, on account of the great quantity of fixed air they evolve in the bowels.—Beer is more or leſs objectionable, according to its ſtrength and imperfect fermentation: he recommends therefore a weak beer, ſomewhat bitter, and well fermented, which is a refreſhing and excellent beverage to quench thirſt. All ſorts of ardent ſpirits are highly pernicious; while tea, coffee, and chocolate, are hurtful only when uſed immoderately, that is, either too hot, or too ſtrong; Bohea tea is, in his opinion, the moſt wholeſome.

With reſpect to dreſs and exerciſe, he gives ſeveral uſeful precepts, and concludes this intereſting and popular treatiſe, with the following remarkable declaration: " I have here advanced no other propoſition, than ſuch as are principally confirmed by my own experience, and of the truth of which

I am

I am fully convinced. I have not publifhed thefe fheets as a Doctor and Profeffor of medicine, but as a convalefcent from hypochondriafis, and for the benefit of my fellow-fufferers, as well as the *candidates for the gout.* If they are ufeful to the public, I fhall, without reluctance, forfeit the approbation of my learned colleagues."

Without wifhing to detract from the merit of originality, equally due perhaps to Mr. Tode, as well as others, we fhall in this refpect obferve, that about twenty years ago, Dr. WEIKARD, of Heilbron (one of the moft zealous apoftles Dr. BROWN has found in Germany), maintained, "that the morbid matter of the gout is the *proximate caufe* of the hypochondriafis and hyfteria;" fee his '*Mifcellaneous medical Writings*, No. I. *Frankfort*, 1788, 8vo. page 117.' Dr. W. upon this occafion, afferted that "if our nerves have, from a variety of caufes, been debilitated and rendered more irritable, it is then probable, that fome fpecific matter of gout, fcattered through the body, and not yet fixed to any particular part (in other places the author calls it *crude* or *immature* matter), produces irregular motions in the nervous fyftem, in confequence of which we are afflicted with a nervous difeafe (hypochondriafis, hyfteria); whereas our anceftors, as is ftill fometimes obferved among country people, after an irregular mode of living, were punifhed with the gout."

To gratify thofe readers who are anxious to reftore to every author his due, we fhall here infert a paffage from the writings of the ingenious STAHL, who was well acquainted with the alternation *now* fuppofed to take place between the hypochondriac and arthritic affections. That great man expreffes his fentiments on this head in the following words: "Inveni utique per hos 24 annos, imo certe amplius, curiofe inquirendo in *reciprocas confpirationes* arthritico-podagrico hæmorrhorum affectuum non folum tantum numerum, fed quod præcipuum eft, tantam conftantiam et univerfalem quafi veritatem harum rerum, ut hinc merito occafionem fumferim, longe diverfum de his rebus conceptum formandi, quem naturæ confentaneum effe a priori et pofteriori abunde expertus fum. Unde adhuc femel animum ad hiftoricam etiam veritatem *confpirationis* hujufmodi hæmorrhoidalium, ifchiadicorum, nephriticorum, fanguinei mictus et podagricorum *confenfuum* et *reciprocarum collifionem* aut *concurfuum*, omnino advertere cohortor. Neque tamen hoc folum, fed fupereft utique circumftantia adhuc ad hiftoriam ipfam pertinens, nullatenus poftrema dignitate æque atque ordine: nifi quod hunc familiarius ita potius invertat aut turbet, ut nulla habita ratione prioris aut pofterioris vel quolibet tempore, ceu promifcue, minus aufpicato fucceffu fefe exferat. Eft hæc *reciprocatio arthritico-podagricorum* pathematum cum hypochondriafis, impetuofis quantumque affectibus: nempe non fimpliciter fpaftico-

 tenfivis, tenſivis, ſed omnino graviter congeſtoriis ſanguinis, et hoc ipſo viſcerum inflammatoriis, non magis periculoſis, quam ſubinde etiam funeſtis." J. E. STAHLII, *Theoria medica vera*. Hallæ, 1737. Pag. 1033. § XXI. XXII.

It is obvious, from this quotation, that in the ſenſe which he affixed to this alternation, Stahl followed his own ſyſtem. He did not indeed derive hypochondriaſis from an arthritic matter, nor did he conſider this as the proximate cauſe of the diſeaſe; but, according to him, the hypochondriaſis, gout, podagra, and a hundred other morbid phenomena, were *molimina naturæ*, generated by congeſtions and diſorders in the circulation, and in the treatment of which every thing depended on the hæmorrhoidal flux, the great axis round which the whole of his ſyſtem turned. Much truth is doubtleſs implicated in this ſyſtem, which was founded on an accurate obſervation of nature; yet, on the whole, it only proves that a '*Theoria medica vera*' may contain many and conſiderable errors, which, however, we cannot diſcuſs in this place, without treſpaſſing too much on our room, and the patience of our readers.

